# The Prevalence and Clinical Features of Body Dysmorphic Disorder Among Dermatology Patients in the Eastern Province of Saudi Arabia

**DOI:** 10.7759/cureus.42474

**Published:** 2023-07-26

**Authors:** Bayan S Al Shuhayb, Sarah Bukhamsin, Alreem A Albaqshi, Fathia Omer Mohamed

**Affiliations:** 1 College of Medicine, King Faisal University, Al-Ahsa, SAU; 2 Department of Clinical Neurosciences, College of Medicine, King Faisal University, Al-Ahsa, SAU

**Keywords:** bdd, dermatology, dysmorphic, disorder, body

## Abstract

Background and objective

Body dysmorphic disorder (BDD), which affects 1.7% to 2.4% of people worldwide, is usually encountered for the first time by nonpsychiatric physicians. Up to 37% of cases have been documented in dermatology clinics. This study aims to estimate the prevalence of BDD among Saudis attending dermatology clinics because the literature is lacking in this field, especially in the Eastern Province.

Methods

This is a cross-sectional study, conducted in 2023. A total of 412 Saudi Eastern Province residents, aged 18 years and older, were included in the study and given a self-administered web-based questionnaire. The study uses the Body Dysmorphic Disorder Questionnaire as one of its three primary measurements, together with sociodemographic data, and dermatological and previous psychological histories.

Results

A total of 412 participants were enrolled in this study. Of the total sample, 64.5% had more than one skin condition, with the rest having only one one. The most received cosmetic treatment in this study was topical agents. It was estimated that the prevalence of BDD is 9.5% among the studied population. However, it was found that there are no significantly associated factors with the prevalence of BDD.

Conclusions

This study reports a prevalence of 9.5% among people visiting dermatological clinics. The prevalence is alarming, which emphasizes the importance of enhancing the awareness of BDD among dermatologists and developing certain guidelines to identify and refer these patients to mental health professionals.

## Introduction

Body dysmorphic disorder (BDD) is a common psychiatric disorder around the world, affecting 1.7% to 2.4% of the general population. Despite its prevalence, BDD remains underrecognized [1]. This disorder has been defined as a preoccupation with an imagined defect in appearance, with exaggerated concern toward unremarkable physical anomalies by the Diagnostic and Statistical Manual of Mental Disorders, Fourth Edition (DSM-IV). To fit the criteria, the preoccupation must cause clinically significant distress in social, occupational, or other areas of functioning. Most of the patients show repetitive compulsive behaviors to examine, improve, or hide the *defect*. Common examples of these behaviors are repeated mirror checking, comparing with others, excessive grooming (e.g., applying makeup and hairstyling), frequent clothes changing, reassurance seeking, and having a restricted diet [2].

BDD is commonly first encountered by nonpsychiatric physicians. It is estimated to range from 0.7% to 2.4% in general clinics, increasing to 9% to 12% in general dermatology clinics, 8% to 37% in cosmetic dermatology clinics, and 2.9% to 53.6% in cosmetic surgery clinics [1,3]. Furthermore, several studies have been conducted to assess the focus of concern in patients with BDD. One such study, which included 188 patients with BDD, reported that the most frequently affected body parts were skin (65%), hair (55%), nose (39%), eyes (19%), and legs (18%) [4]. All these data support the hypothesis that the first physician to have contact with these patients is most likely to be a dermatologist or a plastic surgeon. The reported numbers differ greatly from study to study, which can be generally attributed to the lack of a formal unified screening method used.

BDD is frequently related to significant morbidity, including suicidal ideation and suicide attempts. In addition, these patients are hard to treat as no treatment can satisfy them; for example in a previous study of 250 adults with BDD, 75% of patients sought nonpsychiatric medical treatments. Of these, 72% of the procedures led to no change and 16% worsened BDD symptoms. They are likely going to request extensive workups, consult numerous physicians, and put dermatologists under huge pressure to prescribe unsuitable and ineffective treatments [5]. Because of that, a diagnosis of BDD is generally considered an important part of the dermatology setting. Other reasons include the risk of violence against physicians and malpractice lawsuits.

In Saudi Arabia, apart from two studies, in which the first one was conducted on 363 patients attending a dermatology clinic in Al-Qassim, Saudi Arabia, in which 18.6% screened positive for BDD [6], and the second study was conducted on 497 patients from the dermatology outpatient clinic at King Khalid University Hospital, Riyadh, Saudi Arabia, in which 14.1 % screened positive for BDD [1], the literature concerning BDD prevalence in dermatology clinics in the Eastern Province of Saudi Arabia is not having enough attention and nearly nonexistent. Therefore, the study aims to quantify the prevalence of BDD among individuals attending dermatology clinics and explore the associated features of patients with this condition. The dermatology specialty was chosen because dermatological complaints are the most common complaint by these patients, which put them in a strategic position to diagnose BDD [2,4].

## Materials and methods

Study type

A community-based retrospective cross-sectional study was conducted in the Eastern Province of Saudi Arabia, from December 1, 2022, to March 1, 2023.

Study population and sample size

The target population for this study included all adult individuals, both Saudis and non-Saudis, in the Eastern region who visited dermatological clinics. A minimum sample of 385 adults was required, as calculated using Raosoft, an electronic sample size calculator, at a 95% level of confidence and a 5% margin of error. The inclusion criteria for this study consisted of all women and men, aged above 18 years, who were either Saudi or non-Saudi and from the Eastern Province, attending dermatology clinics. Additionally, participants were required to have no previous diagnosis of psychiatric diseases.

Data collection tools

Through a self-administered web-based survey, data were collected. Questions were adapted from a questionnaire for BDD based on DSM-IV criteria, and other questions were formulated based on the current author’s experience. 

The three primary areas of the study are as follows: (1) sociodemographic information, including age, gender, marital status, residence, occupation, economic status, and level of education; (2) their dermatological history, which treatment requested afterward, and psychological history; and (3) a questionnaire for BDD based on DSM-IV criteria (Body Dysmorphic Disorder Questionnaire or BDDQ).

In cosmetic dermatology, the questionnaire has 100% sensitivity, 92.3% specificity, a positive predictive value of 70%, and a negative predictive value of 100%. The questionnaire was translated from English to Arabic, which is the primary language of the sample, with the assistance of a certified translator. Before beginning to distribute the questionnaire, dermatology patients, university professors, and healthcare professionals reviewed it several times to ensure its validity, accuracy, and clarity. At the beginning of the survey, a brief description of the purpose of the study was stated and a question to take consent for participation was added. 

The questionnaire was electronically distributed to the community in a Google Forms link via different platforms of social media. It is estimated that at least three to four minutes are required to fill out the form. The questionnaire got distributed among nearly 486 adults in Al-Ahsa. Finally, 412 responses were included and 74 responses got excluded according to the stated criteria.

Statistical analyses

Data analysis was performed using IBM SPSS Statistics for Windows, Version 23.0 (IBM Corp., Armonk, NY, USA). Frequency and percentages were used to display categorical variables. The chi-square test was used to test for the presence of an association between categorical variables. The level of significance was set at 0.05.

## Results

A total of 412 participants were included in the study. Table 1 shows the sociodemographic profile of the participants. As for age, 44.2% (182) of the participants were aged between 18 and 24 years, 19.7% (81) were aged between 25 and 34 years, 17.7% (73) were aged between 35 and 45 years, and 18.4% (76) were older than 45 years. As for gender, 26.9% (111) of the participants were males, while 73.1% (301) of the participants were females. As for the place of residency, 83.3% (343) of the participants were living in a city, 15.8% (65) were living in a village, and 1% (4) were living in the outskirts. As for marital status, 36.2% (149) of the participants were single, 60.9% (251) were married, 2.2% (9) were divorced, and 0.7% (3) were widowed. As for the education level, 1.9% (8) of the participants had primary school education, 3.6% (15) had intermediate school education, 26% (107) had high school education, 62.9% (259) had a bachelor’s degree, 2.7% (11) had higher education (Master's/Ph.D.), and 2.9% (12) had other education levels. As for occupation, 4.4% (18) of the participants were doctors, 1.7% (7) were nurses, 10% (41) were teachers, 1.9% (8) were teachers, 1.9% (8) were engineers, 7.5% (31) had administrative work, 34.2% (141) were students, 26.5% (109) were unemployed, and 13.8% (57) had other occupations. As for the monthly income, 60.4% (249) of the participants had an income less than 5,000 SR, 21.4% (88) had an income between 5,000 and 10,000 SR, 15% (62) had an income between 10,000 and 20,000 SR, and 3.2% (13) had an income more than 20,000 SR.

**Table 1:** Sociodemographic profile of the participants (n = 412). SR, Saudi Riyal

Demographical characteristics	n	%
Age (years)		
18-24	182	44.20
25-34	81	19.70
35-45	73	17.70
>45	76	18.40
Gender		
Male	111	26.90
Female	301	73.10
Place of residency		
City	343	83.30
Village	65	15.80
Outskirt	4	1.00
Marital status		
Single	149	36.20
Married	251	60.90
Divorced	9	2.20
Widowed	3	0.70
Education level		
Primary school	8	1.90
Intermediate school	15	3.60
High school	107	26.00
Bachelor’s degree	259	62.90
Higher education (Master's/Ph.D.)	11	2.70
Other	12	2.90
Occupation		
Doctor	18	4.40
Nurse	7	1.70
Teacher	41	10.00
Engineer	8	1.90
Administrative work	31	7.50
Student	141	34.20
Unemployed	109	26.50
Others	57	13.80
Monthly income (SR)		
<5,000	249	60.40
Between 5,000 and 10,000	88	21.40
Between 10,000 and 200,000	62	15.00
>20,000	13	3.20

**Figure 1:**
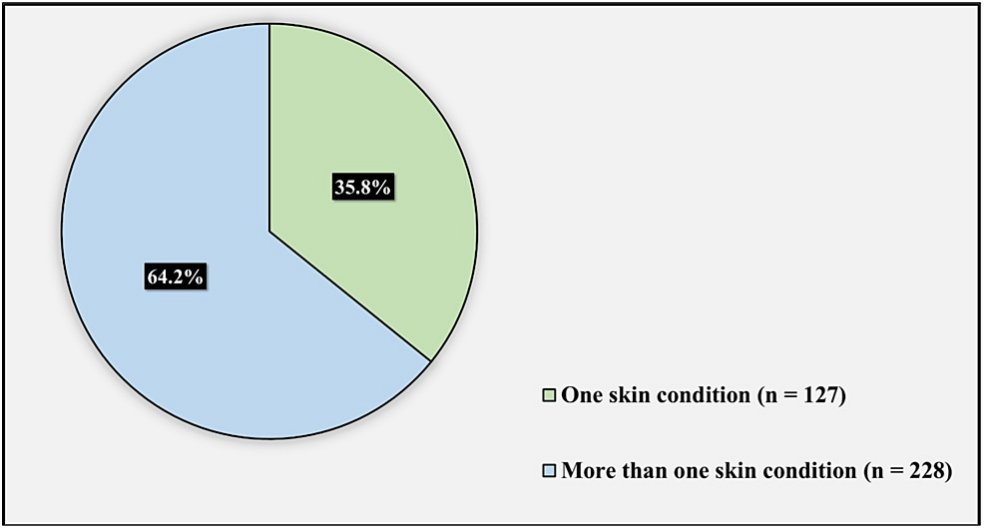
Number of skin conditions affecting the participants.

Figure 1 displays the number of skin conditions affecting participants. Of the 412 participants, 35.8% (127) had one skin condition, while 64.2% (228) had more than one skin condition. 

**Figure 2:**
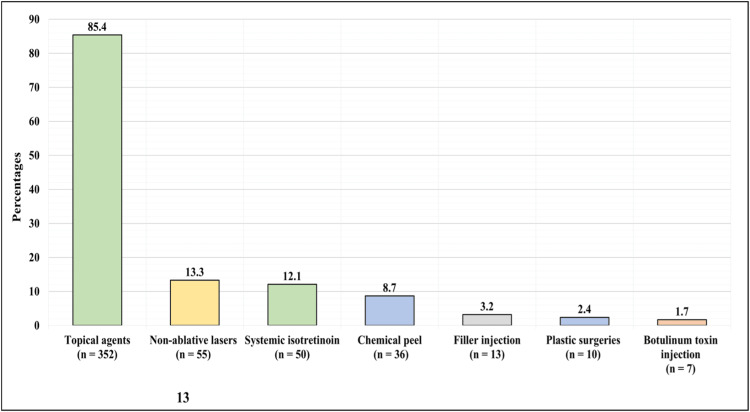
The cosmetic treatment received for the skin conditions.

Figure 2 presents the cosmetic treatment received for the skin conditions. Of the 412 participants, 85.4% (352) received topical agents, 13.3% (55) received non-ablative lasers, 12.1% (50) received systemic isotretinoin, 8.7% (36) received chemical peel, 3.2% (13) had filler injections, 2.4% (10) underwent plastic surgeries, and 1.7% (7) had botulinum toxins injections.

Table 2 demonstrates the assessment of BDD. Of the 412 participants, 38.3% (158) reported worrying about how they looked. Among the 158 participants who were worried about how they look, 93% (147) reported thinking about their appearance problem a lot and wishing they could think of it less. As for the skin conditions that made the participants dislike their appearance, the most commonly reported conditions were hyperpigmentation (45/158, 28.48%), acne (37/158, 23.42%), and stretch marks (22/158, 13.92%). Among the participants who frequently thought about their appearance, 70.1% (103/147) reported spending less than one hour pondering how they look, 20.4% (30/147) spent one to three hours, and 9.5% (14/147) spent more than three hours contemplating their appearance.

**Table 2:** Assessment of body dysmorphic disorder (n = 412).

Question	n	%
Are you worried about how you look?		
Yes	158	38.3
No	254	61.7
Questions directed for those who were worried about how they look (*n* = 158)		
If yes, do you think about your appearance problem a lot and wish you could think of them less?		
Yes	147	93
No	11	7
List the skin conditions that make you dislike your appearance		
Hyperpigmentation	45	28.48
Acne	37	23.42
Stretch marks	22	13.92
Hair loss	21	13.29
Vitiligo	10	6.33
Eczema	7	4.43
Scars	6	3.80
Wrinkles	6	3.80
Psoriasis	4	2.53
Is the cosmetic treatment received in the previous question is for this skin condition?		
Yes	112	70.88
No	46	29.11
Questions directed for those who think about appearance problems a lot (*n* = 147)		
Is your main concern with how you look that you are not thinking enough or that you might get too fat?		
Yes	65	44.2
No	82	55.8
Has it often upset you a lot?		
Yes	76	51.7
No	71	48.3
Does it often get in the way of doing things with friends, dating, your relationships with people, or your social activities?		
Yes	38	25.9
No	109	74.1
Has it caused you any problem with school, work, or other activities?		
Yes	31	21.1
No	116	78.9
Are there things you avoid because of how you look?		
Yes	60	40.8
No	87	59.2
On an average day, how much time do you usually spend thinking about how you look?		
<1 hour	103	70.1
1-3 hours	30	20.4
>3 hours	14	9.5

**Figure 3:**
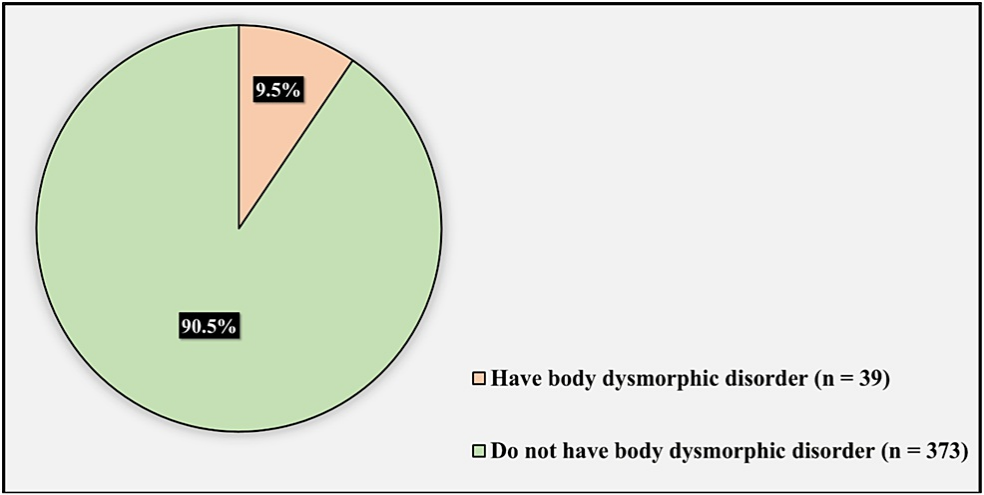
The prevalence of body dysmorphic disorder.

Figure 3 illustrates the prevalence of BDD. The prevalence of BDD was 9.5%, affecting 39 participants, while 373 individuals (90.5%) did not have BDD.

Table 3 shows the factors associated with the prevalence of BDD. Age, gender, place of residency, marital status, education level, occupation, monthly income, and several skin conditions affected the participants. As for the individuals who fulfilled the criteria of BDD, 76.9% were females, and slightly less than half of the affected individuals, 48.7%, were aged from 18 to 24 years, followed by 25.6% aged between 25 and 34 years. As for the place of residency, the majority of the participants (82%) were living in the city and only 15% were living in villages. The majority of the affected participants were married (58.9%), with the rest being single or divorced. As for the education level, nearly 64.1% of the affected participants had a Bachelor’s degree, followed by 25.6% having a high school degree, with the rest having intermediate and primary school degrees and higher education Master's/Ph.D. degrees. As for the occupation, nearly half of the affected participants (46.1%) were students, followed by 15.3% being unemployed. As for the monthly income, slightly more than half of the affected participants (53.8%) had an income less than 5,000 SR, followed by 30% having an income between 5,000 and 10,000 SR. Nearly half of the affected individuals (53.8%) reported having more than one skin condition, with the rest reporting only one condition.

**Table 3:** Factors associated with the prevalence of BDD. ^*^Significant at level 0.05. BDD, body dysmorphic disorder

Factor	Prevalence of BDD, *n* (%)		*P*-value^*^
	Have BDD	Don't have BDD	
Age (years)			0.213
18-24	19 (10.4)	163 (89.6)	
25-34	7 (8.6)	74 (91.4)	
35-45	10 (13.7)	63 (86.3)	
>45	3 (3.9)	73 (96.1)	
Gender			0.567
Male	9 (8.1)	102 (91.9)	
Female	30 (10)	271 (90)	
Place of residency			0.980
City	32 (9.3)	311 (90.7)	
Village	6 (9.2)	59 (90.8)	
Marital status			0.944
Single	15 (10.1)	134 (89.9)	
Married	23 (9.2)	228 (90.8)	
Divorced	1 (11.1)	8 (88.9)	
Education level			0.994
Primary school	1 (12.5)	7 (87.5)	
Intermediate school	1 (6.7)	14 (93.3)	
High school	10 (9.3)	97 (90.7)	
Bachelor’s degree	25 (9.7)	234 (90.3)	
Higher education (Master's/Ph.D.)	1 (9.1)	10 (90.9)	
Occupation			0.301
Doctor	3 (16.7)	15 (83.3)	
Nurse	0 (0)	7 (100)	
Teacher	3 (7.3)	38 (92.7)	
Engineer	0 (0)	8 (100)	
Administrative work	2 (6.5)	29 (93.5)	
Student	18 (12.8)	123 (87.2)	
Unemployed	6 (5.5)	50 (87.7)	
Monthly income (SR)			0.515
<5,000	21 (8.4)	228 (91.6)	
Between 5,000 and 10,000	12 (13.6)	76 (86.4)	
Between 10,000 and 200,000	5 (8.1)	57 (91.9)	
>20,000	1 (7.7)	12 (92.3)	
Number of skin conditions affecting participants			0.583
One skin condition	13 (8.4)	141 (91.6)	
More than one skin condition	26 (10.1)	232 (89.9)	

## Discussion

A psychiatric condition, known as BDD, is defined by excessive anxiety about a small or nonexistent physical imperfection to the extent that it interferes with the patient's ability to lead a normal life. It is distinct from the common uncertainties and self-doubts that everyone encounters occasionally in that it is more intense and has a crippling impact on the patient's social and professional life [6].

Previous studies have found BDD to be common in approximately 1.7% to 2.4% of the general population and even higher (4.4%) among Saudi females [1]. For cosmetic dermatological clinics, BDD is reported with a frequency as high as 53.6% [7-10]. Regarding the general dermatology outpatient clinics, about 7.9% screened positive for BDD in Germany, 14% in America, 18.6% in Al-Qassim, and 14.1% in Riyadh [1,6,11-13]. The Saudi prevalence is lower in the current study (9.5%), which can be explained by the limited sample size in this study. Moreover, both studies, which were done in Saudi Arabia, did not exclude patients previously diagnosed with mental illness, explaining the higher prevalence.

Different epidemiological studies showing different levels of BDD prevalence can be explained by multiple factors. First, body image discrepancies between Arab and Western cultures because of sociocultural differences. Second, the outcomes of various BDD screening tools give different results. However, the BDDQ that is used in the current study has been shown by a study that 89% of the patients screened positive for BDD with BDDQ were also diagnosed with BDD using the Structured Clinical Interview for DSM-IV (SCID) [14]. Third, different study population characteristics have an impact on BDD results. For instance, the relatively alarming prevalence of BDD in general dermatology patients in our current study (9.5%) may be because more patients had BDD-related conditions like acne and hyperpigmentation. Although body dysmorphic disorder symptoms typically start in early childhood, a formal diagnosis might take 10 to 15 years. This was supported by the study done in Al-Qassim, in which BDD significantly decreased with age and was highly expressed in students [6,11]. This might be explained by the excessive use of social media by young people exposes them to unrealistic standards of beauty and aesthetics. In this study, the majority of the patients (48.7%) who screened positive for BDD were aged 18 to 24 years. Although most previous studies showed that BDD is more prevalent among women, some reports showed no correlation between gender and BDD diagnosis, supported by the results of the current study [1,6,7]. All sociodemographic characteristics and BDD did not appear to be correlated in this research. 

Regarding dermatological conditions, the majority who screened positive reported hyperpigmentation as the most concerning problem followed by acne and hair loss. This is supported by two studies done in Riyadh and Brazil [1,5]. This can be explained by the fact that people with ethnic skin are expected to experience considerable psychological effects from hyperpigmentation. Most of the previous studies showed that having more than one skin condition is correlated with BDD [1,6,7]. Patients with multiple conditions are more mindful of other small skin or bodily abnormalities, leading them to be more self-conscious, and the behavioral characteristics that patients with BDD have. In the current study, nearly 53% of participants who screened positive had more than one skin condition. 

One of the findings of the current study is that 25% (10) of the patients who screened positive for BDD did not seek dermatological treatment for their BDD-related concerns. That comes in line with two previous studies. This has been explained by the authors it might be related to the shame of disclosing BDD concerns to medical providers. Another justification is that people with BDD spend an excessive amount of time looking at their skin. Therefore, they are more likely to notice any changes more quickly than those without BDD [1,15]. Thus, this study leads to the conclusion that BDD is a concealed, underdiagnosed, and untreated disease.

This study had some limitations. To reach a larger scale, a self-administered survey that was distributed through several social media platforms was used for data collection. Second, the scale used is a screening tool for BDD; hence, the prevalence estimate may be higher than it is. Third, this study lacks an objective evaluation of patient’s concerns, meaning that patients with moderate and severe physical defects were included in the count. Fourth, because the Saudi healthcare system only offers medical dermatology treatments and does not offer specialized cosmetic dermatology, this study focused only on the general dermatology population. Since it has been widely documented that BDD prevalence varies across general dermatology and cosmetic dermatology settings, this might have skewed the results.

## Conclusions

This study reports a prevalence of 9.5% among people visiting dermatological clinics. The prevalence is alarming, which emphasizes the importance of enhancing the awareness of BDD among dermatologists and developing certain guidelines to identify and refer these patients to mental health professionals. Patients with BDD may not be satisfied with any dermatological treatment provided. In some cases, this can lead to self-harm or even suicide or individuals seeking unnecessary appearing-enhancing procedures. Healthcare providers need to take the concerns of patients with BDD seriously, refer them for psychiatric treatment, and recommend appropriate care for this condition.
